# Natural Resources – Food Nexus: Food-Related Environmental Footprints in the Mediterranean Countries

**DOI:** 10.3389/fnut.2014.00023

**Published:** 2014-12-12

**Authors:** Cosimo Lacirignola, Roberto Capone, Philipp Debs, Hamid El Bilali, Francesco Bottalico

**Affiliations:** ^1^International Centre for Advanced Mediterranean Agronomic Studies (CIHEAM), Mediterranean Agronomic Institute of Bari, Bari, Italy

**Keywords:** natural resources, environmental footprints, dietary patterns, Mediterranean region, water footprint

## Abstract

Immediate action is required in the Mediterranean to address environmental degradation that is mainly driven by consumption patterns. Increasing stress on biological and social systems is put by unsustainable consumption patterns. Food consumption patterns are important drivers of environment degradation. The objective of this review paper is to explore natural resources-food nexus in the Mediterranean region by highlighting the environmental footprints of the current consumption and production patterns. Secondary data from different sources such as FAOSTAT, the World Bank, Water Footprint Network (WFN), and Global Footprint Network were used to analyze the situation in 21 Mediterranean countries. The region faces many environmental challenges, e.g., land degradation, water scarcity, environment pollution, biodiversity loss, and climate change. The current consumption patterns imply high ecological, carbon, and water footprints of consumption and unfavorable national virtual-water balances. Food Balance Sheets data show that the contribution of vegetal and animal-based food product groups to food supply is variable among the Mediterranean countries. This has implications also in terms of the WF of food supply, which was calculated for Bosnia, Egypt, Italy, Morocco, and Turkey. The WF of the current diet resulted lower than that of the proposed Mediterranean one in the case of Italy. There is a strong scientific evidence supporting assumption that it is so also for other Mediterranean countries. The Mediterranean is characterized by a high resource use intensity that is further exacerbated by food losses and waste (FLW). In fact, FLW implies the loss of precious resources (water, land, energy) and inputs (fertilizers). Therefore, it is crucial to increase adherence to the traditional Mediterranean diet and to reduce FLW in order to foster transition to more sustainable food consumption patterns thus reducing pressure on the scarce resources of the Mediterranean region.

## Introduction

In the Mediterranean, immediate action is required to address environmental degradation that is mainly driven by population and consumption. Increasing stress on biological as well as social systems is put by unsustainable consumption patterns, in particular food consumption patterns that are important drivers of environment degradation, e.g., unsustainable water use, declining soil fertility, marine environment degradation, biodiversity loss, climate change (CC), etc. Much of today’s discourse about environmental problems revolves around reducing greenhouse gas (GHG) emissions and water usage.

Mediterranean region’s development cannot be “sustainable” except if the fundamental common goods are protected and improved. Protection of the coast, sea, climate and air quality, soil and biodiversity, water resources, cultural and landscape heritage, and traditional knowledge of nature are the priorities to be focused on. It is very important to break the joints that make economic development reliant on an intensive exploitation of natural resources and to promote changes in consumption and production patterns (1).

In this context, the current food system delivers low cost food at a high cost to the environment (2) and this cost includes also environmental impacts of food production, distribution and consumption (3). As a very important factor in critical sustainability issues (4) diets affect different factors (social, cultural, agricultural, environmental, nutritional, and economic) which interact with one another. In fact, in the Mediterranean region, many issues (water, biodiversity loss, scarcity, soil erosion, etc.) are linked to food consumption patterns, and it should be addressed as priorities (5). There are signs that diet has an impact on health (4), but the sustainability of food consumption and food systems regards also environmental impacts.

If no changes are implemented in the coming years, there is a high risk of further deterioration of the global food system with consequent degradation of the environment and the natural resource base. This alarming trend can jeopardize the capacity of the worldwide ecosystems to generate enough resources, especially food, to feed the growing world population within the planetary natural limits. Another issue that should be highlighted the externalities of the current food systems. As a matter of fact, food production patterns release huge amounts of GHG and other toxic pollutants (6). Sustainable diets have been defined by FAO and Biodiversity (7) as those that ensure food for future generations without compromising the natural resources and the environment (7). These diets are based on locally produced food that is available, affordable, nutritious, and safe. Furthermore, they keep the farmers’ incomes, cultures of consumers, and local communities’ lifestyles and traditions. A sustainable diet puts food, biodiversity and nutrition at the core of sustainable development and the right to food.

The Mediterranean Diet (MD), in fact, is widely recognized as a healthy dietary pattern. Furthermore, it was inscribed by UNESCO on the Representative List of intangible cultural heritage of humanity. For these reasons the MD, in its diversity, was chosen by the Food and Agriculture Organization of the United Nations (FAO) as its first case study to develop a methodology for diets sustainability assessment [e.g., Ref. (7–10)]. In fact, the importance of the MD as a case study is due not only to its specific foods and nutrients but also to the sustainability philosophy that is one of the most important features of this lifestyle pattern (11).

Adherence to the MD has been linked to health benefits and considerable nutrition (12–17).

Thus, MDs, to be considered sustainable, should respect and protect biodiversity and ecosystems, have low environmental impact, and optimize natural resources.

Recommendations for lowering GHG emissions and energy inputs from household food consumption include diets with more locally produced and fresh foods and less meat and dairy products and more in-season vegetables (18–20).

So, it is important to stress how sustainability, diets, food security, and water are closely connected. With urbanization and rising incomes, typical dietary patterns are shifting toward consumption patterns based on animal products (21, 22) requiring more water, land resources, and energy (23, 24).

In the Mediterranean region, there are at least four main environmental challenges related to the current Mediterranean production and consumption patterns that should be addressed: water scarcity, land degradation, CC, and biodiversity loss.

The first constraint in the Mediterranean area is represented by *water scarcity* that represents the most critical development problem and by consequence it is the main limit for agricultural growth. In Mediterranean region, since the late 1950s water availability has been declining steadily. By opposite, during the second half of the twentieth century, water demand has doubled and agriculture is the main water-consuming sector accounting for 64% of total water demand. The irrigated land accounts for 20% of all arable land and produces 40% of food production. Half of the “water poor” world population is concentrated in the Southern Mediterranean region (25) and it has been estimated that by 2025 potentially 180 million people will be affected by water problems (26). Demographic pressures, together with the economic development of non-agricultural sectors, will further deteriorate water balance in many Mediterranean countries where the water exploitation index is already a matter of serious concern.

The second constraint concerns the various forms of *land degradation*, particularly erosion (27). Land degradation is as old as the region but new threats have appeared in modern times in connection with the economic and social upheavals of recent years, poor farming intensification in certain sectors, urban and industrial waste pollution, encroachment on space by urbanization and infrastructures, and so on. The Mediterranean region possesses about 854 million ha of total land, but only 118 million ha of them are suitable for agricultural production. Contrary to tropical countries, options for agricultural expansion are extremely limited and if land is reclaimed for agriculture, costs are high and the newly reclaimed soils result of poor quality needing further investments to keep their productivity.

Land degradation in the form of salinization, water and wind erosion, sand encroachment, compaction, organic matter decline, sealing, and coastal *littoralization* are severe in many Mediterranean countries. Soil salinization and alkalization are forms of soil degradation that represent the major causes of desertification in the Mediterranean (28). Human-induced salinization has expanded mostly due to poor quality irrigation water and irrigation management, especially along the coasts where seawater intrusion into the fresh water aquifers is a common problem. While analyzing the status of land resources, particular attention should be given to agriculture land. In the Mediterranean EU countries, the average agricultural land per capita is 0.30 ha and the agricultural land per agricultural worker is 11.4 ha, while in the Middle East and North Africa (MENA) countries (including Turkey) the first value is 0.25 ha and the second is only 1.9 ha, indicating that land available for agriculture is much less. Other indicators link population increase with availability of agricultural land. It is estimated that in 2020, compared with 1961, the Mediterranean population would more than double while agricultural land area will shrink by losing 8.3 million ha (7%) if the actual rates of urbanization and land degradation will remain the same (29). Consequently, the agricultural land (ha/capita) region wide would drop from 0.48 ha in 1961 to 0.21 ha in 2020. Considering that the MENA region would have more than 300 million people in 2020 this last ratio becomes particularly relevant.

Another important aspect treated in this paper concerns *CC*. In fact, in the Mediterranean region, it is affecting food security and agriculture in the region mainly through changes in precipitation, temperature, sea level rise, and extreme climatic events (30). Furthermore, CC may affect deterioration of land degradation water scarcity, crop failures, fisheries production, livestock deaths, and quality decline. In the Southern and Eastern Mediterranean countries (SEMCs), desertification is one of the biggest constraints to productivity. In fact, in the dry areas people mainly depend on natural resources and on agriculture for their livelihood and desertification hits them hard. So, CC refers to any change in climate over time which, triggered with other expected and plausible changes (e.g., population growth and migrations, social, economic and technological development, political, financial and cultural setup, consumption and living habits, dietary preferences) will create new scenarios that will affect the availability and quality of water and land resources used in agricultural production and the biodiversity of ecosystems. Therefore, the Mediterranean might be a particularly vulnerable region to CC and especially in the areas already characterized by water scarcity and land degradation. In fact, the warming trend and changes in precipitation pattern might further affect the water balance and composition and functioning of natural and managed ecosystems. In particular, CC impacts on agriculture could be relevant with interrelated effects on the biophysical factors (physiological effects on forests, crops, pasture, and livestock; changes in water resources, soil and land; increased weed, and pest challenges, etc.) and socio-economic impacts (changes in yields and food production, fluctuations in world market prices, etc.). Moreover, besides the changes in food availability, the collateral effects of CC could be expected over the whole chain of food system stability, accessibility, and utilization, including the water and energy used in food processing, storage, and transport, as well as the consideration of environmental services (31) and on biodiversity.

In fact, as for *biodiversity*, in the Mediterranean basin, there is wide climatic, geographic, and topographic variability resulting in an enormous range of habitat diversity and species. The region scores third in biodiversity richness at world level (32) as it hosts 30% of endemic fauna and 60% of all unique flora species, about 8% of the known marine species and with 30,000 plant species (33). The Mediterranean Sea, in fact, contains 8–9% of all marine species in the world (34). Its geo-morphological and geological history and the position of the biomes from temperate to tropical, enable it to accommodate both species affinities hot and cold (35) and to host a strong proportion of all endemic species (over 25%).

The importance of the Mediterranean is stressed by the fact that Mediterranean region provides about one-third of the foodstuffs used by humankind (36). Wheat, barley, oats, grapes, olives, figs, almonds, peas, dates, and other huge amount of fruits and vegetables as well as aromatic herbs or medicinal derived from wild plants are found in the Mediterranean region (34). But changes in diet in the Mediterranean region are having an impact on biodiversity.

The first main cause of reduction of biodiversity is the habitat loss and/or fragmentation and the factors contributing to habitat loss are: land use competition, overpopulation, deforestation, pollution (air, water, soil), and global warming due to CC. Many Mediterranean lagoons and deltas are disappearing and for surface coastal ecosystems, the most serious threat is posed by the construction of facilities and coastal *artificialization*. Such typology of “urbanization” leads to the loss of ecosystems with a high level of biodiversity (35). The other main cause of biodiversity reduction and loss is the natural resources over-exploitation. In the case of forests or pastures, there is currently a huge disparity between the situations prevailing on the two banks of the Mediterranean (37). To the north, biodiversity is at risk in areas of extreme farm intensification and increasing urbanization. The nature of the pressure in the south of the Mediterranean region is different as there is still very strong over-exploitation of forests and shrubs for firewood. Also the over-exploitation of the Mediterranean marine biodiversity currently appears to be one of the major threats to fish, in particular to the migratory ones and to some mollusk, sea urchin, and shellfish species. CC appears to encourage the geographic spread of the exotic invasive species in the Mediterranean Sea. Little is known about the possible impact of CC on many marine species, but as a consequence of it many aquatic permanent and ephemeral ecosystem might disappear (35).

Biodiversity is closely associated to agriculture. It is clear that CC will induce changes in agricultural areas suitable for cultivation of specific crops, especially those that are characteristic of Mediterranean area such as olive. CC may increase temperature and changes in rainfall regimes anticipate both an increase and decrease in precipitation, and an increased frequency of dry spells and floods (38). These changes will impact both rainfed and irrigated agriculture. According to Jarvis et al. (39) CC will increase the genetic erosion of landraces and threatening wild species including crop wild relatives.

Given the above-described environmental challenges, the objective of this review paper is to explore natural resources – food nexus in the Mediterranean region by highlighting the environmental footprints of the current consumption and production patterns. In fact, it is assumed that only by decreasing the environmental impacts of the current food consumption patterns, a concrete transition to a more environmentally sustainable food system can be fostered in the area. Indeed, environment is one of the most important pillars of sustainability, therefore, a sustainable food system should rely mostly on the regional domestic biocapacity and natural resource base.

## Materials and Methods

The review paper is based on secondary data from different databases and sources such as FAOSTAT, World Development Indicators (WDI) of the World Bank, Water Footprint Network (WFN), Global Footprint Network (GFN), UNEP/MAP-Plan Bleu, European Commission (DG ENV), CIHEAM (International Centre for Advanced Mediterranean Agronomic Studies), Barilla Centre for Food & Nutrition (BCFN), Critical Ecosystem Partnership Fund (CEPF), Intergovernmental Panel on Climate Change (IPCC), Italian Institute of Food Science (La Sapienza university), Stockholm International Water Institute, World Resources Institute, WWF, etc.

The geographical coverage of this study is similar to that of the Mediterranean Strategy for Sustainable Development (1) including 11 Northern Mediterranean Countries (Albania, Bosnia and Herzegovina, Cyprus, Spain, France, Greece, Croatia, Italy, Montenegro, Malta, and Slovenia) and 10 SEMCs (Algeria, Egypt, Israel, Lebanon, Libya, Morocco, Palestinian territories, Syria, Tunisia, and Turkey). The UE-28 Mediterranean countries gather eight countries: Croatia, Cyprus, Spain, France, Greece, Italy, Malta, and Slovenia. In addition to these countries data were collected and analyzed as well for Portugal, Serbia and Macedonia (FYROM).

Standard impact data (per kilogram or metric ton of food product and/or food group) were used to calculate and discuss the environmental impacts and footprints of food consumption patterns. This paper focus on ecological footprint (EF), carbon footprint (CF), and water footprint (WF) of consumption.

The EF method allows knowing how much of the biosphere regenerative capacity is occupied by human-related activities (40). Regenerative capacity or *biocapacity* refers to the capacity of ecosystems to produce useful biological materials and to absorb waste generated by human activities (41). Ewing et al. (42, 43) described the methodology for the calculation of the EF on a national scale. The EF measures *biocapacity* (in global average bio-productive hectares) across six major land use types: cropland, fishing grounds, grazing land, forest land, CF, and built-up land. The EF methodology uses a consumer-based approach to keep track of both direct and indirect *biocapacity* needed to support consumption patterns. The EF of consumption (EFC) is calculated for each land use type as: EFC = EF of production + EFI-EFE; where EFI and EFE refer to the EFs embodied in imported (EFI) and exported (EFE) commodities. Comparison was made between the following regions: Middle East (Egypt, Jordan, Lebanon, Occupied Palestinian Territory, Syria, Turkey), North Africa (Algeria, Libya, Morocco, Tunisia), Northern Mediterranean countries (Cyprus, France, Greece, Italy, Malta, Portugal, Slovenia, Spain), Central and Northern Europe (Austria, Belarus, Belgium, Denmark, Estonia, Finland, Germany, Iceland, Ireland, UK), and North America (Canada and USA).

The CF is a measure of the exclusive total amount of CO_2_ emission directly and indirectly caused by an activity or accumulated over the life stages of a product (44). In the methodology of the GFN, the CF as well is expressed in global hectares. Carbon dioxide emissions are the only waste product included in the National Footprint Accounts. The CF is calculated as the amount of forest land required to absorb given carbon emissions. In particular, the CF is the total amount of anthropogenic CO_2_ emissions (e.g., land use change, deforestation, etc.) minus the amount of CO_2_ absorbed by oceans in a given year translated into the amount of bio-productive forest that would be needed to store it that year (42).

As for the WF, definitions of the Global Water Footprint Standard of the WFN are used (45). The WF of a product is similar to what has been called alternatively product’s embedded, embodied, exogenous water; “virtual-water content” of a product or shadow water (46). The WF represents a measure of human’s appropriation of freshwater resources and refers to water resources required to produce goods and services. Measurement of freshwater appropriation takes into consideration volumes of water consumed (evaporated or embodied into a product) as well as that polluted per unit of time (47). The WF concept takes into account the use of *blue water* (surface and ground water), *green water* (moisture stored in soil strata and rain water), and *gray water*. The last is defined as the volume of freshwater that is required to assimilate the load of pollutants given existing water quality standards and natural background concentrations (45). The WF of consumption (WFcons) of a country is the sum of direct and indirect use of domestic and foreign freshwater resources to produce the goods consumed by its inhabitants (48). Secondary data from the WFN were used to analyze WFs of consumption and virtual-water balances in the Mediterranean countries.

*Water footprints of food supply* were calculated for five Mediterranean countries: Bosnia, Egypt, Italy, Morocco, and Turkey. These countries were selected as representatives of different Mediterranean macro-regions: Italy (Northern Mediterranean), Egypt and Turkey (Eastern Mediterranean), Bosnia (Balkans), and Morocco (Southern Mediterranean). The methodology used in the present paper for the calculation of the WF of national food supply is similar to that used by Sáez Almendros et al. (49) for the analysis of the Spanish dietary pattern environmental footprint. WFs of animal products as well as crops and derived crop products were obtained from Mekonnen and Hoekstra (50, 51). WF of food supply in each country was calculated using average WF per ton of commodity per country, weighted based on food product origin (47). Adopting a consumption perspective, the paper identifies the consumed food products driving pressure on water resources in the five Mediterranean countries and makes comparison with Northern Europe (Finland) and North America (USA). The main problems faced were related to the management and processing of data thus some simplification was necessary. There were also some difficulties about the availability of data regarding footprints of some food products or food product groups.

Food supply data from the Food Balance Sheets (FAO-FBS) were used to characterize the *Mediterranean dietary patterns* (MDPs). Data regarding food consumption patterns in the USA and Finland, that exemplify the western dietary patterns (WD), were obtained as well from the FAO-FBS (52). According to Zessner et al. (53), the conversion of food product supply values (as given by the FAO-FBS) to actual consumption values implies two correction factors: the first factor accounts for food components not eaten and the second for food waste and feed to domestic animals. The *share of vegetal-based components in total energy consumption* represents the contribution, in terms of energy supply, of the following food groups: cereals (excluding beer), starchy roots, *sugarcrops*, sugar and sweeteners, pulses, *treenuts*, *oilcrops*, vegetable oils, vegetables, fruits (excluding wine), stimulants, spices, alcoholic beverages, and aquatic products (other than fish and seafood). Meanwhile, the *share of animal-based energy in total dietary energy* reflects, in relative terms, the energy supply of the following food groups: meat, offals, animal fats, eggs, milk (excluding butter), fish, and seafood.

Environmental cost, in terms of water use, of non-adherences to the Mediterranean dietary pattern was analyzed in the Italian context by comparing the WF of the MD and that of the current dietary pattern in Italy. The dietary composition of the MD in the Italian context was obtained from diet proposed by the Italian Institute of Food Science of the La Sapienza University (54). Two independent data sources were used to estimate the current Italian diet: FAO-FBS and the Italian Food Consumption Survey 2005–2006 carried out by the Italian National Institute of Research for Food and Nutrition (55).

The paper analyses also fertilizer and mineral nitrogen consumption, as proxy indicators for resource use intensity related to food consumption patterns. *Fertilizer consumption* measures the quantity of plant nutrients used per unit of arable land. Fertilizer products cover nitrogenous, potash, and phosphate fertilizers (56). Data are available for the period 2002–2009 from the WDI. *Mineral nitrogen consumption* accounts for nitrogen input that implies the use of nitrogen fertilizers in agricultural production. It is calculated as the average quantity of mineral nitrogen (in kilogram) used per hectare of national agricultural land. Data are available for the period 2002–2010 from FAOSTAT – Resources database.

## Results and Discussion

### Ecological and carbon footprints of consumption

Regarding the *EF of production*, the resources used in 2007 by Mediterranean countries need, in order to regenerate, a period that range from 1 year and 3 months to 5 years and 5 months in Albania and Libya, respectively. Regarding the EFC, the period that is needed to regenerate the resources consumed ranges from 1 year and 6 months to 8 years and 6 months in Croatia and Jordan, respectively. Therefore, the Mediterranean countries have a net demand greater than their respective biocapacity: expressed in average values, 2 years and 3 months are needed to regenerate the resources used for production, whereas 3 years and 4 months are required to regenerate the resources that are effectively consumed.

**Figure 1:**
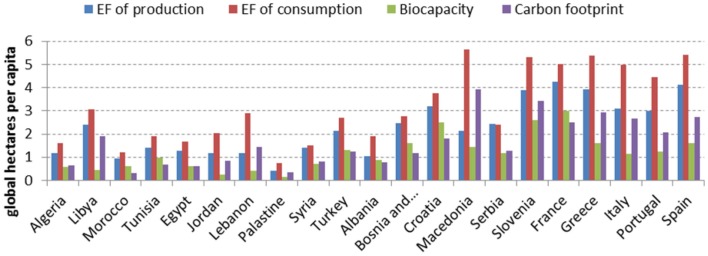
**EF of production, EF of consumption, *Biocapacity*, and Carbon footprint in the Mediterranean region [Source: adapted from Ref. (42)]**.

As shown in Figure 1, the EFC in the Mediterranean are always higher than the EF of production, except for the case of Serbia. The CF alone is generally higher than the *biocapacity*, except for Albania, Bosnia, Croatia, France, Morocco, Tunisia, and Turkey.

Generally speaking, the northern Mediterranean countries present a higher EF with respect to North Africa and Middle East ones. EFs of production and consumption and CF of North American countries are higher than those recorded in Mediterranean countries even the Northern ones. In the Mediterranean context, the northern region present higher CF (mainly Greece and Spain) and EF of production and consumption (mainly Spain, Greece, and France) with respect to North Africa and Middle East regions (Table 1).

**Table 1:** **Ecological footprints of production and of consumption, *biocapacity*, and carbon footprint (in global hectares per capita) in the Mediterranean countries**.

Geographical areas	Nation	EF of production	EF of consumption	*Biocapacity*	Carbon footprint
North Africa	Algeria	1.18	1.59	0.59	0.63
	Libya	2.4	3.05	0.44	1.92
	Morocco	0.93	1.22	0.61	0.33
	Tunisia	1.42	1.9	0.98	0.68
Middle East	Egypt	1.29	1.66	0.62	0.62
	Jordan	1.18	2.05	0.24	0.83
	Lebanon	1.18	2.9	0.4	1.43
	Palestine	0.4	0.74	0.16	0.34
	Syria	1.4	1.52	0.7	0.8
	Turkey	2.13	2.7	1.32	1.24
Balkan area	Albania	1.05	1.91	0.87	0.77
	Bosnia-Herzegovina	2.47	2.75	1.6	1.17
	Croatia	3.21	3.75	2.5	1.81
	Macedonia	2.12	5.66	1.43	3.94
	Serbia	2.44	2.39	1.16	1.27
	Slovenia	3.88	5.3	2.61	3.42
North Mediterranean	France	4.27	5.01	3	2.51
	Greece	3.94	5.39	1.62	2.92
	Italy	3.08	4.99	1.14	2.66
	Portugal	2.99	4.47	1.25	2.07
	Spain	4.13	5.42	1.61	2.73
North America	North America	8.39	7.9	4.93	5.42

*Source: adapted from Ref. (42)*.

**Figure 2:**
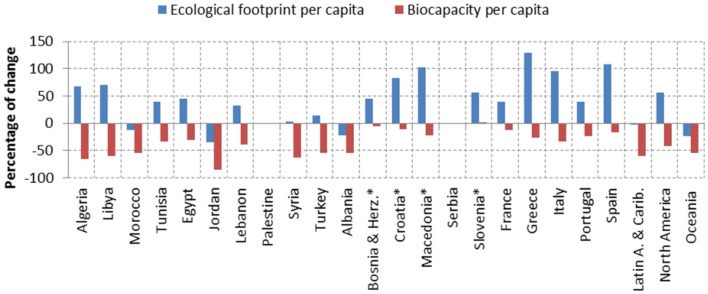
Evolution of the EF and biocapacity in the Mediterranean countries from 1961 to 2007 (*data shows change between 1992 and 2007) [Source: adapted from Ref. (42)].

In the period 1961–2007, the EF per capita in the Mediterranean has increased except in Albania, Jordan, and Morocco, while the *biocapacity* has decreased as shown in Figure 2, thus the ecological deficit increased. On average, the EF has increased by 47.4% while the *biocapacity* has decreased by 36.4%.

**Figure 3:**
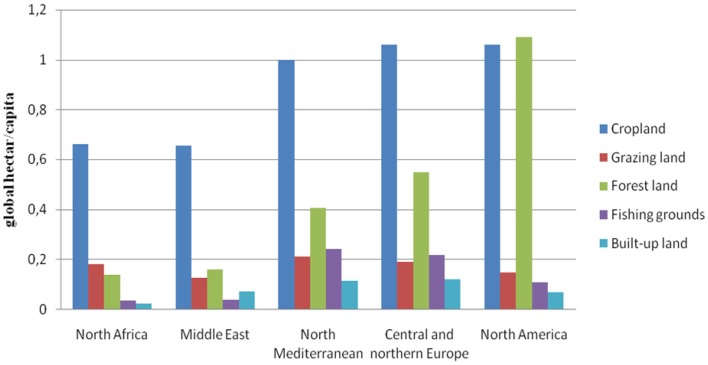
**Ecological footprint (in global hectares per capita) by land use type in the Mediterranean [Source: adapted from Ref. (42)]**.

Taking into consideration land use types (cropland, grazing land, forestland, fishing grounds, and built-up land), Figure 3 shows that the EF of cropland is highest in North Mediterranean countries and in central and northern Europe, while the EF of forestland is highest in North America. The average EF in Northern Mediterranean countries is at least 1.5 times the EF of North Africa and the Middle East. The fact that cropland EF is the highest in the Mediterranean highlights the relevance of food production (agriculture) and consumption patterns in terms of land use in the region. It implies that moving to more sustainable dietary patterns in the Mediterranean can have positive effects in terms of pressure reduction on land resources with a consequent decrease of the ecological debt and deficit of the countries of the region especially the northern Mediterranean ones.

### Water footprint of consumption and virtual-water balance

**Figure 4:**
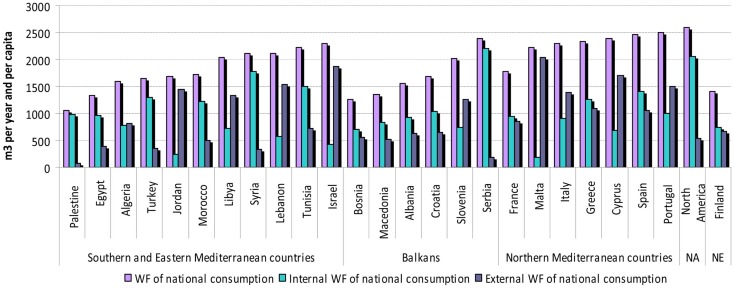
**Water footprint of national consumption [Source: adapted from Ref. (47)]**. NA, North America; NE, North Europe.

From 1996 to 2005, WF of consumption varies widely among Mediterranean countries as shown in Figure 4, in particular the internal and external WF of consumption. In fact, the share of the external WF of consumption ranged from 7.3 to 91.8%, in Palestine and Malta, respectively. The WF of national consumption ranges between 1055 m^3^/year/capita in Palestine and 2505 m^3^/year/capita in Portugal. Northern Mediterranean countries present higher WF of consumption compared to SEMC and the Balkan countries. The WF per capita in the Mediterranean, especially in SEMC, are lower than in North America but higher than the WF of consumption of Finnish citizens.

**Figure 5:**
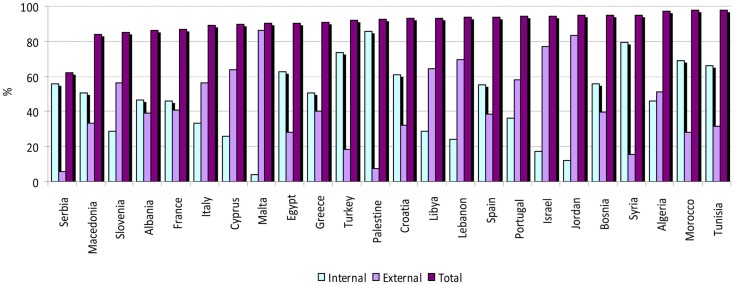
**WF of agricultural products consumption in Mediterranean countries [Source: adapted from Ref. (47)]**.

Most of the WF of consumption is due to the consumption of agricultural products (Figure 5). The share of the WF of agricultural products consumption in the total WF of consumption ranges from 61.8% in Serbia to 97.7% in Tunisia. The average rate is about 91% of the total WF of consumption.

Only Tunisia, Serbia, and Syria, present a negative total net *virtual-water balance* (Table 2). The other Mediterranean countries present a positive net virtual-water balance. The main reason is that most of Mediterranean countries are not self-sufficient for many products so they import them. Doing so, they import also virtual water. The other Mediterranean countries show water savings that range from 340 Mm^3^ to 62,157 Mm^3^, in Macedonia and Italy, respectively. This is due to the fact that the production of agricultural/industrial goods is very water efficient in NMC as compared to the other SEMC, i.e., virtual-water contents of goods are relatively lower.

**Table 2:** **Net virtual-water balance (in million cubic meters per year)**.

Regions	Countries	Total net virtual-water balance (green + blue + gray)
SEMC	Algeria	17,311
	Egypt	9,051
	Israel	7,411
	Jordan	5,667
	Lebanon	4,057
	Libya	9,559
	Morocco	8,337
	Syria	−2,267
	Tunisia	−1,666
	Turkey	5,786
Balkan countries	Albania	1,165
	Bosnia and Herzegovina	1,891
	Croatia	1,973
	Macedonia	340
	Serbia	−1,780
	Slovenia	1,415
NMC	Cyprus	1,173
	France	12,822
	Greece	6,903
	Italy	62,157
	Malta	529
	Portugal	10,246
	Spain	24,203

*Source: adapted from Ref. (47)*.

### Water footprint of food supply

#### Food supply in the Mediterranean region

According to FAO Food balance sheets (57), the dietary energy in the Mediterranean, in 2009, ranged between 2130 kcal/day/person in Palestine and 3666 kcal/day/person in Turkey. Generally speaking, in northern Mediterranean countries, the dietary energy is higher. FAO Food Balance Sheets show that dietary energy increased in all the SEMCs in the period 1990–2009, except in Turkey, Libya, and the Palestinian territories. In the Mediterranean, the share of plant-based energy in the diet is usually higher than 50%. In general, it is higher in eastern and southern Mediterranean countries with respect to northern ones, while intermediate values are recorded in the Balkan countries. The share of plant-based energy in the diet is higher in the Mediterranean than in Northern Europe and America. Taking into consideration 2009 data, the shares of vegetal-based energy in the diet is 66.5% in France and 88.8% in the Palestinian Territories. The largest share of plant-based energy is derived from cereals. In general, that share is higher than in northern Europe and North American (e.g., USA). Moreover, the contribution of vegetal-based products to the total dietary energy decreased between 1990 and 2009 in most of the Mediterranean countries (Table 3).

**Table 3:** **Changes of dietary energy and share of vegetal-based energy in the diet in the Mediterranean in the period 1990–2009**.

Country	1990		2009		Change in the period 1990–2009 (%)	
	Food supply (Kcal/capita/day)	Vegetal products (%)	Food supply (Kcal/capita/day)	Vegetal products (Kcal/capita/day)	Food supply	contribution of vegetal-based products to the total dietary energy
Albania	2656	82.38	2903	70.34	9.3	−12.04
Algeria	2855	88.83	3239	89.10	13.5	0.27
Bosnia (1992)^a^	2419	88.84	3070	82.67	26.9	−6.17
Croatia (1992)^a^	2412	76.24	3130	73.19	29.8	−3.05
Cyprus	2685	72.70	2678	73.97	−0.003	1.27
Egypt	3154	93.34	3349	91.40	6.2	−1.94
France	3515	62.02	3531	66.50	0.5	4.48
Greece	3539	78.89	3661	76.70	3.4	−2.19
Israel	3398	80.69	3569	78.29	0.05	−2.41
Italy	3584	73.91	3627	74.28	1.2	0.36
Lebanon	2965	86.17	3153	82.97	6.3	−3.20
Libya	3222	86.56	3157	87.58	−2.0	1.02
Macedonia (1992)^a^	2418	81.64	2957	81.50	22.3	−0.14
Malta	3078	74.72	3438	73.59	0.12	−1.13
Montenegro (2006)^a^	2681	71.54	2887	72.19	7.7	0.65
Morocco	3073	93.20	3264	91.54	6.2	−1.65
Palestinian Territories (1996)^a^	2321	87.72	2130	88.87	−8.2	1.15
Portugal	3393	76.66	3617	70.86	0.07	−5.80
Serbia (2006)^a^	2696	77.11	2823	78.07	4.7	0.96
Slovenia (1992)^a^	2670	73.90	3275	71.42	0.23	−2.48
Spain	3279	74.69	3239	74.50	−1.2	−0.19
Syria	2896	87.15	3212	85.62	10.9	−1.54
Tunisia	3124	91.33	3314	89.50	6.1	−1.83
Turkey	3766	89.03	3666	88.38	−2.7	−0.65

*^a^In case data for 1990 are not available the reference year is put in brackets after the country name*.

*Source: FAO Food Balance Sheets (57)*.

According to Vanham et al. (58), for a healthy diet in the EU28 (EU27 and Croatia), including Northern Mediterranean countries, the intake of some product groups should be reduced (i.e., crop oils, sugar, animal fats, and meat), and the intake of other product groups like vegetables and fruit should be increased.

In Italy, dairy products are the largely consumed foods, while in Egypt and Morocco, cereals are the most consumed food item. Whereas, a different situation exists in Bosnia and Turkey, where vegetables are the most consumed food products. This has implications in terms of WFs of food consumption.

#### Water footprint of food supply in Italy, Bosnia, Morocco, Egypt, and Turkey

Among the five considered Mediterranean countries, the lowest WFs of food supply are recorded in Egypt (1194.70 m^3^/capita/year) and Turkey (1291.65 m^3^/capita/year) while the highest is recorded in Bosnia (1849.70 m^3^/capita/year), which is slightly higher than the WF recorded in Italy (1848.29 m^3^/capita/year) and in Morocco (1644.85 m^3^/capita/year). The average WF of an Italian citizen is 35.36, 30.12, 11.01% higher and 0.08% lower than that of an Egyptian, a Turkish, a Moroccan, and a Bosnian one, respectively. Regarding the Southern Mediterranean countries, the average WF in Morocco is 37.68% higher than the one recorded in Egypt. The total WF of food supply in the USA (2198.66 m^3^/capita/year) is higher than in the other five Mediterranean countries while the Finnish WF (1116.69 m^3^/capita/year) is lower.

According to Vanham et al. (58) and Vanham and Bidoglio (48), the total current WF of consumption in EU28 is 4815 liters/capita/day (lcd) (i.e., 1757.47 m^3^/capita/year). Of the latter 40% is external to Europe. The WF of agricultural products contributes the largest fraction, about 89% of the total WF of consumption (48). Edible products account for the largest fraction of the total WFcons, i.e., 4032 lcd (1471.7 m^3^/capita/year). This shows that by changing the diets the WFcons can be largely reduced (58).

From a country to another, the shares of the three components of the WF (green, gray, and blue) change. In all the Mediterranean countries, except for Egypt (green: 40.3%, gray: 17.7%, blue: 42.0%), the highest WF is the green one, followed by the gray for Bosnia (green: 88.0%, gray: 9.0%, blue: 3.0%) and Italy (green: 84.0%, gray: 8.8%, blue: 7.2%), and the blue one in the case of Morocco (green: 83.4%, gray: 4.5%, blue: 12.1%) and Turkey (green: 80.6%, gray: 8.2%, blue: 11.2%). As for Egypt, the first component is the blue one while the green one is ranked second. The reason why the highest share of the blue water component in the total WF is recorded in Egypt is mainly due to the fact that water is used in irrigation.

Meat products’ contribution to the total WF is the highest in Bosnia and Italy where about a third of the total WF is due to the consumption of meat products. While the contribution of cereals to the total WF is the highest in SEMCs (Egypt, Morocco, and Turkey), where they account for more than a third of virtual-water use. The contribution of vegetable oils (e.g., olive oil) to the WF is relevant in Italy but not in the other countries. When considering both dairy products and meat, they represent more than a half of the total WF of food supply in Bosnia and Italy (Table 4).

**Table 4:** **Contribution of food product groups to the total water footprint of food supply in Bosnia, Italy, Turkey, Morocco, and Egypt; 2006**.

Food products	Bosnia	Italy	Turkey	Morocco	Egypt
Meat	31.81	39.62	16.57	27.28	31.51
Vegetable oils	4.01	14.54	7.35	5.68	1.81
Milk	21.92	12.22	12.56	7.60	6.29
Cereals	9.13	11.01	40.07	37.56	33.42
Stimulants	10.03	6.84	2.15	2.78	0.40
Fruit	5.97	3.62	4.49	4.92	6.80
Sugar and sweeteners	4.57	3.10	3.12	2.56	3.85
Alcoholic beverages	2.30	1.99	0.39	0.20	0.14
Animal fats	2.18	1.88	1.00	2.53	1.39
Vegetables	2.47	1.87	3.48	1.88	5.31
Offals	0.84	1.01	0.54	0.99	2.40
Others	4.78	2.31	8.30	6.03	6.68
Eggs	0.31	0.80	3.06	2.87	0.86
Oil crops	0.11	0.56	2.36	0.31	1.89
Pulses	2.02	0.46	2.00	2.05	1.78
Starchy roots	0.04	0.42	0.60	0.49	0.85
Spices	0.00	0.07	0.29	0.30	0.66
Sugar crops	0.00	0.00	0.00	0.00	0.64

The food product with greatest impact is the red meat, while fruit and vegetables have definitely limited footprints (59–61). Generally speaking, when the consumption of animal products is lower (especially beef meat) environmental impact is also lower. Meat production generates high environmental impacts (62–64). According to Vanham et al. (58), the consumption of animal products accounts for high WF amounts.

Intensive livestock production system has several negative impacts on the planet’s resources and ecosystems, and for this reason it is necessary to switch to a more resource-efficient and healthier vegetable-rich diet (48). At local and global level, the livestock industry is one of the largest contributors to environmental degradation (65, 66).

With traditional water use statistics, awareness campaigns and policy have always focused on increasing water efficiency in domestic and industrial water use. However, by reducing food waste and by changing the diet, much more water can be saved in agricultural production processes (48).

The top 10 products contributing to the total WF of food supply change from a country to another (Table 5). Wheat is the first of the list in the case of Egypt, Morocco, and Turkey, while the top products are bovine meat for Italy and milk for Bosnia. In the case of Italy, the top 10 products are, in descending order: bovine meat, milk, wheat, coffee, poultry meat, cocoa beans, sunflower seed oil, offals, potatoes, and maize.

**Table 5:** **Top ten contributing products to food supply water footprint (%) in Italy and Turkey; 2006**.

Items	Italy	Items	Turkey
Bovine meat	14.0	Wheat	37.0
Milk	12.2	Milk	12.6
Wheat	10.2	Bovine meat	8.0
Coffee	5.1	Poultry meat	5.9
Poultry meat	1.9	Sunflower seed oil	1.8
Cocoa beans	1.7	Maize	1.1
Sunflower seed oil	1.3	Coffee	0.9
Offals	1.0	Potatoes	0.6
Potatoes	0.4	Offals	0.5
Maize	0.2	Cocoa beans	0.5

### Water footprint of the current and proposed food consumption patterns in Italy

The total water of the diet proposed by the Italian Institute of Food Science of La Sapienza University is 964.29 m^3^/capita/year: 79.10% green, 11.46% blue, and 9.44% gray. Fruit and meat are the most significant contributors to the total *WF of the proposed diet*. These two food product groups represent about 50% of the total WF of the proposed Italian diet. Each of milk, extra-virgin olive oil, meat cuts, and bread represents more than 5% of the total WF. The seven previously mentioned food products represent about 80% of the proposed diet WF (Table 6).

**Table 6:** **Contribution of different food products to the total water footprint of the proposed diet in Italy**.

Products	Total water footprint (m^3^/capita/year)	%
Fruits	262.57	27.23
Meats	215.27	22.32
Extra-virgin olive oil	94.93	9.84
Meat cuts	78.53	8.14
Milk	52.30	5.42
Bread	51.02	5.29
Vegetables	43.64	4.53
Pasta	35.22	3.65
Cheese	28.23	2.93
Yogurt	23.62	2.45
Rice	14.20	1.47
Butter	11.05	1.15
Juice	10.66	1.11
Cookies	10.27	1.07
Pulses	9.75	1.01
Eggs	8.39	0.87
Sugar	6.72	0.70
Potatoes	4.53	0.47
Salad	2.01	0.21
Honey	1.39	0.14

The contribution of different food product groups to *water footprint of the current Italian dietary pattern* changes depending on the component that is taken into consideration, i.e., green, blue, or gray (Table 7). Bakery products and cereals are the main contributors to the total WF; actually, they contribute more than two-fifths to the WF of the current Italian food consumption pattern. Combined with pulses, these products represent more than 50% of the total WF. While fruits and vegetables – the most consumed food groups – contribute less than a fifth to the total WF.

**Table 7:** **Contribution of different food product groups to the green, blue, and gray water footprints of the current dietary pattern in Italy**.

	Total water footprint (m^3^/capita/year)		
	Green	Blue	Gray
Cereals and bakery products	587.95	49.32	48.52
Pulses, fresh and preserved	297.73	19.81	31.63
Vegetables	155.34	5.66	19.41
Potatoes	110.82	8.30	1.06
Fruit	79.63	9.87	12.96
Meat	68.77	3.66	4.62
Milk, derivatives and substitutes	32.56	8.47	5.53
Oils and fats	26.22	5.70	3.89
Alcoholic beverages and substitutes	14.38	1.15	3.14
Confectionery (sweets) and substitutes	8.33	0.86	1.25
Eggs	5.14	0.09	0.92
Water and soft drinks	2.12	0.72	0.78
Others	1.68	0.11	0.19
Total	1390.68	113.72	133.90

The current Italian food consumption pattern presents a WF (1638.30 m^3^/capita/year: green: 84.9%, gray: 8.2%, blue: 6.9%), that is about 70% (i.e., 674 m^3^/capita/year) higher than that of the proposed diet (964.29 m^3^/capita/year: green: 79.1%, gray: 9.4%, blue: 11.5%).

That means that a full adherence to the proposed MD for the last 6 years (from 2006 to the end of 2011) would have allowed saving more than 152,749 million cubic meters of water (Table 8).

**Table 8:** **Water saving assuming a 100% adherence of the Italian population to the proposed Mediterranean diet during the last 6 years (from 2006 to the end of 2011) (in million cubic meters – Mm^3^)**.

Years	2006	2007	2008	2009	2010	2011	Total water saving (Mm^3^)
Italian adult population (18–64 years) (ISTAT data^b^)	37,250,394	37,523,477	37,760,955	37,906,233	38,095,091	38,095,091	^a^
Water saving by the Italian population (Mm^3^/year)	25,106.8	25,290.8	25,450.9	25,548.8	25,676.1	25,676.1	152,749.5

*^a^Work hypothesis: It is assumed that the food consumption pattern has not changed meanwhile so that the same annual amount of water is saved per person*.

*^b^Population at January 1st was considered that of the previous year. Data referring to January 1st, 2011, were considered for 2010 and 2011*.

This water saving represents about 294 times the blue domestic water consumption in Italy in 2005 (47). In other words, the estimated water saving, generated by the adult Italian population’s adherence to the proposed MD, is enough to cover domestic water consumption for almost 294 years in Italy.

Taking into consideration that the average total abstraction of freshwater in Italy is around 42 km^3^/year (67), this water saving represents total water abstraction (including household, industry, agriculture, and energy water demands) for more than 3 and 4 years.

Vanham et al. (58) showed that different EU28 diets – a healthy, combined, and vegetarian diet – as compared to the current average diet would substantially reduce the EU28 WFcons for agricultural products. Of the diets analyzed, the vegetarian one present the lowest WFcons. Meat intake reduction has the biggest impact on the reduction of the WF due to the high WF per caloric value of meat products.

### Fertilizer consumption and nitrogen fertilizers use trends

Fertilizer consumption indicator gives an idea about the level of intensification of agricultural production as it assesses input use intensity. Fertilizers can have detrimental impacts on natural ecosystems if they are not properly and rationally used.

During the period 2002–2009, fertilizers consumption ranged from 6.0 kg/ha of arable land recorded in Algeria (2003) to 696.6 kg/ha recorded in Egypt (2008). In the same period, average fertilizers consumption ranged between 11.6 kg/ha recorded in Algeria and 563.0 kg/ha recorded in Egypt. Fertilizer consumption in Egypt is even 4.5 higher than that recorded in the European Union. That can be explained by the fact that almost the whole arable land in Egypt is irrigated and agriculture is intensive. In the period 2002–2009, the average fertilizers consumption in the 21 target Mediterranean countries was 188.0 kg/ha, so higher than the worldwide average (116.3 kg/ha of arable land). During the same period, the average fertilizers consumption in the Middle East & North Africa (90.6 kg/ha) was lower than the levels of fertilizers consumption in the Euro area (179.9 kg/ha) and the European Union countries (155.5 kg/ha).

Considering the period 2002–2009, fertilizers consumption decreased in almost all target Mediterranean countries. In fact, it increased only in Egypt (70.3%), Cyprus (22.3%), Montenegro (11.3%), Tunisia (18.3%), and Turkey (23.7%). The highest decrease was recorded in Lebanon (−188.9%) and Slovenia (−161.6%).

In the period 2002–2010, mineral nitrogen consumption ranged between 0.1 kg/ha recorded in Algeria in 2005 and 468.9 kg of mineral nitrogen/ha of agricultural land recorded in Egypt in 2003. In the Mediterranean region are used on average 52.9 kg N/ha. During the same period, the highest average nitrogen use was recorded in Egypt (373.0 kg N/ha) while the lowest was recorded in Algeria (0.9 kg N/ha) (Table 9).

**Table 9:** **Mineral nitrogen consumption in some Mediterranean countries (kilograms of mineral nitrogen/ha of agricultural land)**.

Country	2002	2003	2004	2005	2006	2007	2008	2009	2010
Albania	33.3	33.1	31.9	34.2	26.8	27.7	23.8	27.9	25.7
Algeria	0.7	0.4	2.5	0.1	1.1	0.9	0.6	0.5	1.1
Bosnia	8.8	5.0	15.5	11.4	7.2	10.0	5.5	10.7	8.4
Croatia	109.9	103.3	123.7	103.1	155.4	176.7	204.4	135.0	50.1
Egypt	312.5	468.9	396.4	416.7	293.9	312.7	441.2	326.8	388.2
France	74.5	80.0	78.5	74.6	74.7	81.7	71.6	65.2	70.3
Greece	32.2	62.2	27.0	51.3	20.9	29.1	21.3	16.1	17.9
Israel	79.4	91.1	102.0	94.8	93.4	114.6	89.0	69.1	58.4
Italy	55.3	56.8	58.2	54.3	56.2	57.4	46.4	34.9	34.8
Lebanon	31.9	26.8	27.4	13.4	11.8	19.5	14.7	19.0	23.6
Libya	4.9	2.1	3.0	4.8	2.4	4.4	2.7	3.0	3.0
Malta	59.8	50.1	64.3	62.2	100.7	61.4	36.5	41.9	32.3
Montenegro	−	−	−	−	3.4	2.7	2.6	2.1	2.7
Morocco	8.2	6.7	7.7	11.2	10.7	10.2	9.2	6.5	4.5
Portugal	42.6	28.9	33.0	26.9	23.2	30.3	25.7	26.8	34.4
Slovenia	65.3	66.7	60.9	56.7	61.0	58.7	50.6	59.9	56.1
Spain	34.9	41.1	36.9	31.7	33.9	35.2	26.3	27.9	34.2
Syrian	16.1	17.5	16.3	19.3	19.9	18.9	19.4	15.1	8.6
Tunisia	3.8	5.4	4.9	6.3	6.6	4.0	5.9	7.8	6.9
Turkey	29.1	33.0	33.2	33.3	34.7	34.4	29.0	36.3	34.4
*Mediterranean*	*54.5*	*61.8*	*57.2*	*56.4*	*50.0*	*53.7*	*54.7*	*44.7*	*43.4*

*Source: Authors’ elaboration based on data from FAOSTAT*.

In the period 2002–2010, average mineral nitrogen consumption decreased in the Mediterranean area from 54.5 to 43.4 kg of mineral nitrogen/ha of agricultural land. Mineral nitrogen consumption decreased in all Mediterranean countries except in Algeria (+0.4 kg N/ha), Tunisia (+3.1 kg N/ha), Turkey (+5.3 kg N/ha), and Egypt (+75.7 kg N/ha). The highest decrease was recorded in Croatia (−59.8 kg N/ha).

Mineral nitrogen trade balance (export – import) is negative in the Mediterranean area. Considering the period 2002–2010, mineral nitrogen trade deficit increased from −1,275,809 to −1,441,037 tonnes of nitrogen, i.e., +165,228 N tonnes. As of 2010, almost all the Mediterranean countries are net mineral nitrogen importers except Israel, Jordan, Tunisia, Croatia, Libya, Morocco, and Egypt. The top net mineral nitrogen exporters are Egypt (1,381,065 tonnes of nitrogen) and Morocco (327,023 tonnes of nitrogen). Meanwhile, the top net mineral nitrogen importers are France (−1635 N tonnes), Turkey (−967175 N tonnes), Italy (−409540 N tonnes), Spain (−369243 N tonnes), and Greece (−124780 N tonnes).

High fertilizers, especially nitrogen ones, use calls in question the environmental sustainability of the current Mediterranean food consumption patterns.

### Environmental implications of food losses and waste

Food losses and waste (FLW) reduction is now considered as essential to reduce the environmental footprint of food systems (52, 68–72). In fact, this is presented as crucial for reducing the emission of GHGs, thus slowing the pace of CC, and des-intensifying natural resources use.

The long food products journey involves consumption of resources, labor, and, consequently, of GHG emissions. So, when considering a foodstuff throughout its life cycle, one must also take into account the water, energy, and resources consumed, and, therefore, wasted when this food becomes refuse (73).

Food losses and waste have two major direct environmental impacts: waste of the resources that are used to produce the food lost and wasted (use of 1.4 billion hectares of arable land) and major source of negative impacts including emissions of GHG at disposal (3.3 billion tons of GHGs released into the atmosphere) (74, 75). Indirect environmental externalities include unnecessary ground and surface water pollution caused by the intensive use of nitrogenous fertilizers in agriculture. FLW negative externalities include also those that agriculture expansion into wild areas and mono-cropping create in terms of loss of biodiversity (74). Approximately, one-third of food produced for human consumption that is lost or wasted globally every year is estimated to be equivalent to 6–10% of human-generated GHG emissions (76). A recent study estimated that food waste in the EU27 generates about 170 Mt of CO_2_ eq.each year (77).

Many recent scientific studies have attempted to analyze environmental impacts of FLW usually addressing categories such as GHG emissions (CF) [e.g., Ref. (78)], WF [e.g., Ref. (48, 79, 80)], nitrogen [e.g., Ref. (81)], and land use (EF) [e.g., Ref. (82)].

Food loss and waste account for more than 25% of the total consumptive use of vulnerable and limited freshwater resources and more than 300 million barrels of oil per year (22, 83). Globally, the blue WF of food wastage is about 250 km^3^ (74, 75). Minimizing waste can reduce water demand; a decrease about 50% in FLW at the global level would save 1,350 km^3^ a year (52).

Most of the WF of consumption in the Mediterranean countries is due to the agricultural products’ consumption. The share of the WF of agricultural products consumption in the total WFcons ranges from 61.8% in Serbia to 97.7% in Tunisia. The average value is approximately 91% of the total WF of consumption (47). Taking into account the WF of agricultural products consumption (47), considering the conservative FLW percentage of 30% [cf. Ref. (84)] and assuming that the same amount of water is wasted whenever food is lost and/or wasted [cf. WWF (85)], it can be realized that from 294 (Palestinian territories) to 706 m^3^/capita/year (Portugal) of water are lost or wasted by Mediterranean people (Table 10).

**Table 10:** **Estimates of water losses and wastage in the Mediterranean countries**.

Mediterranean regions	Countries	WF of consumption (m^3^/capita/year)	WF of agricultural products consumption (%)	Water losses and wastage due to food losses and waste (m^3^/capita/year)
Southern and Eastern Mediterranean countries	Palestine	1055	93	294
	Egypt	1341	90	364
	Turkey	1642	92	453
	Algeria	1589	97	463
	Jordan	1678	95	478
	Morocco	1725	98	505
	Libya	2038	93	571
	Lebanon	2112	94	593
	Syria	2107	95	600
	Tunisia	2217	98	650
	Israel	2303	94	650
Balkans	Macedonia	1348	84	340
	Bosnia	1256	95	358
	Albania	1555	86	401
	Serbia	2390	62	443
	Croatia	1688	93	471
	Slovenia	2012	85	513
Northern Mediterranean countries	France	1786	87	466
	Malta	2216	90	599
	Italy	2303	89	617
	Greece	2338	91	636
	Cyprus	2385	89	640
	Spain	2461	94	692
	Portugal	2505	94	706

*Source: Authors’ calculation based on data from Mekonnen and Hoekstra (47)*.

Reducing the quantity of food required, by reducing waste, could mitigate the negative effects of land use change, and CO_2_ emissions from agriculture (86). Making the food chain more efficient through waste reduction measures will reduce pressure on resources required for food production and lower GHG emissions (6). In fact, reducing the amount of food wasted throughout the food chain in the entire Mediterranean area would help improving food and nutrition security and contribute to easing pressure on natural resources especially water. Reducing waste across the whole food system will increase the amount of food available for human consumption for the given level of inputs, thereby improving input use efficiency (3). Reducing food loss and wastage will reduce water needs in agriculture (22) as well as environmental impacts (22, 87). Interventions to reduce waste in the food chain will have a greater impact on availability of freshwater resource as other measures of water use efficiency in agriculture and food production (88).

## Conclusion

References linking food and environment can be found going back millennia. Food and nutrition has always straddled sectors such as agriculture and environment. Recent formalized manifestations include sustainable consumption and production and sustainable diets.

For addressing the challenge of feeding the growing Mediterranean population, especially in southern Mediterranean countries, new strategies to ensure food and nutrition security while allowing natural resources conservation are required. Population increase, industrial development, globalization, and urbanization have dramatically affected Mediterranean food production and consumption patterns with impacts on natural ecosystems as well as diets. Consequent trends in terms of human health and ecosystem integrity degradation are alarming. The present food system is unsustainable and is putting increasing stress on ecosystems – in terms of the supply of resources, goods, and services – and human social systems. Food consumption patterns are considered important drivers of environmental pressures and footprints. Food production systems, i.e., agriculture sector (including crop production, fisheries, and animal production), holds much of the blame for environment problems (loss of agro-biodiversity, water resources depletion, groundwater and soil contamination, land degradation, GHG emissions, etc.).

For fostering and speeding up transition toward more sustainable food consumption patterns profound changes in both food consumption and food production are necessary. Efforts relating to the promotion of sustainable agriculture should be complemented by consumption-related measures. In fact, developing a sustainable food system requires transformative and simultaneous interventions covering all phases of the food chain, i.e., from field to fork. It also requires unprecedented, large-scale behavior change. Sustainability in food systems means addressing coherently and simultaneously the consumptive demand and productive supply elements by fostering smarter and efficient food production systems and diets.

Improving food consumption patterns in the Mediterranean region implies minimizing pressure on natural resource and externalities over the life cycle. Sustainable food consumption encompasses sustainable diets, water consumption decrease, waste reduction, sustainable supply chains, and energy use efficiency improvement. Preference should be given for diets that have low environmental impacts, but provide the required amount of nutrients (including micronutrients) and energy for a healthy life and a sustainable lifestyle. These diets should be based on local, seasonally produced, minimally processed, ecologically packed, and tasteful foods.

Pressure on the scarce natural resources, especially water, is expected to increase due to demographic changes in SEMCs. Food demand increase will have effects on volumes of irrigation water. Nowadays, almost 65% of Mediterranean freshwater resources are used in irrigated agriculture. The ecological deficit and debt of the Mediterranean region will further increase in the coming years as the EF per capita is increasing while the *biocapacity*, i.e., the regenerative capacity of Mediterranean ecosystems, is decreasing.

Resource use intensity is still high in Mediterranean countries. The average fertilizers consumption in the Mediterranean countries is higher than the worldwide average. Mineral nitrogen consumption remains high in many Mediterranean countries especially those that rely on intensive irrigated agriculture (e.g., Egypt). The Mediterranean region is a net mineral nitrogen importer.

The WF of consumption varies widely amongst Mediterranean countries. Nevertheless, more than 90% of the total WF of consumption in the Mediterranean region is due to agricultural products consumption. Meat, dairy products, and wheat represent more than a half of the WF of food supply in Mediterranean countries. The contribution of meat and dairy products is higher in northern Mediterranean countries with respect SEMCs. Cereals supply accounts for more than a third of the WF of food supply in some SEMCs such as Egypt, Morocco, and Turkey.

The enhancement and promotion of the MD is crucial for food consumption environmental sustainability. MD promotion measures can contribute to sustainable natural resources management. The Italian case study shows clearly that a significant water saving can be yielded thanks to adherence to the Mediterranean dietary pattern. In fact, the WF of the current Italian dietary pattern is much higher than that of the suggested MD.

It is important, furthermore, to reduce food wasted throughout the food chain to make the Mediterranean food system more sustainable. For this reason it is of paramount importance to alert consumers to the environmental implications of their food-related behavior (e.g., diets, overeating, wasting food). FLW reduction can ease pressure on natural resources and free up water resources and land for other development purposes, societal needs and economic sectors.

Transition toward a sustainable food system in the Mediterranean region requires developing integrated, coherent, comprehensive and holistic policies dealing with nutrition, health, agriculture, environment and natural resources, economy, society, and culture. Multifaceted actions and interventions should be implemented as a package to yield the desired sustainable changes. Policies and actions at all levels require more and better *intersectoral* research to simultaneously address food and environmental sustainability. In particular, it is important to assess the environmental sustainability of the Mediterranean food consumption patterns taking into account the different environmental footprints (e.g., CF, nitrogen footprint, WF, EF, energy footprint). Food waste is another issue that should be taken into account for assessing more accurately the environmental sustainability of diets.

These activities require a shared regional multi-actor governance model involving policy makers, academia, and scientific community as well as representatives of farmers, the food industry, the civil society and consumer organizations. Coordinated actions are needed at all levels (local, national, and regional). Local multi-actor partnerships and initiatives to achieve sustainable food systems can be scaled up within sound regional regulatory frameworks.

## Conflict of Interest Statement

The authors declare that the research was conducted in the absence of any commercial or financial relationships that could be construed as a potential conflict of interest.
